# *MnFtz-f1* Is Required for Molting and Ovulation of the Oriental River Prawn *Macrobrachium nipponense*

**DOI:** 10.3389/fendo.2021.798577

**Published:** 2021-12-20

**Authors:** Huwei Yuan, Wenyi Zhang, Yin Fu, Sufei Jiang, Yiwei Xiong, Shuhua Zhai, Yongsheng Gong, Hui Qiao, Hongtuo Fu, Yan Wu

**Affiliations:** ^1^ Wuxi Fisheries College, Nanjing Agricultural University, Wuxi, China; ^2^ Key Laboratory of Freshwater Fisheries and Germplasm Resources Utilization, Ministry of Agriculture, Freshwater Fisheries Research Center, Chinese Academy of Fishery Sciences, Wuxi, China; ^3^ East China Sea Fisheries Research Institute, Chinese Academy of Fishery Sciences, Shanghai, China

**Keywords:** 20E, *Macrobrachium nipponense*, MnFtz-f1, RNAi, molt, ovulation

## Abstract

Molting and ovulation are the basic processes responsible for the growth and reproduction of *Macrobrachium nipponense*; however, the molecular mechanisms of molting and ovulation in *M. nipponense* are poorly understood. The present study aimed to use *MnFtz-f1* as the starting point to study the molting and ovulation phenomena in *M. nipponense* at the molecular level. The full-length *MnFtz-f1* cDNA sequence was 2,198 base pairs (bp) in length with an open reading frame of 1,899 bp encoding 632 amino acids. Quantitative real-time PCR analysis showed that *MnFtz-f1* was highly expressed in the ovary at the cleavage stage and on the fifth day after hatching. *In vivo* administration of 20-hydroxyecdysone (20E) showed that 20E effectively inhibited the expression of the *MnFtz-f1* gene, and the silencing of the *MnFtz-f1* gene reduced the content of 20E in the ovary. *In situ* hybridization (ISH) analysis revealed the localization of *MnFtz-f1* in the ovary. Silencing of *MnFtz-f1* by RNA interference (RNAi) resulted in significant inhibition of the expression of the vitellogenin (*Vg*), *Spook*, and *Phantom* genes, thus confirming that *MnFtz-f1* had a mutual regulatory relationship with *Vg*, *Spook*, and *Phantom*. After RNAi, the molting frequency and ovulation number of *M. nipponense* decreased significantly, which demonstrated that *MnFtz-f1* played a pivotal role in the process of molting and ovulation.

## Introduction

Molting is an important behavior in the growth and development of arthropods. A growing body of evidence shows that 20-hydroxyecdysone (20E) controls or triggers the molting process in arthropods, and the uncoordinated action of 20E is often fatal (1–5). The molting process of arthropods requires multilevel regulation, which involves some members of the nuclear receptor family of genes that perform important functions in the molting process (6). The synthesized 20E binds to the nuclear receptor genes to regulate downstream genes and jointly regulate molting (7). Thus, nuclear receptor-type transcription factors are essential for the molting process of arthropods (6).

Nuclear receptors are a family of transcription factors characterized by a central DNA binding region (8). The average insect has 21 genes encoding nuclear receptors (9). In-depth research has been conducted on the role of nuclear receptors in life activities of insects, such as oogenesis, embryonic development, and molting (9, 10). The nuclear receptor *Ftz-f1*, as the potential factor of molting response, plays a central role in coordinating different molting processes (11, 12). *Ftz-f1* is induced after the level of 20E decreases (13–15). In *Nilaparvata lugens*, 20E was found to significantly inhibit the expression of *Ftz-f1*, indicating that *Ftz-f1* was directly regulated by 20E (16). One isoform of *Ftz-f1* has been detected in most insects such as *Bombyx mori* (17), *Aedes aegypti* (18), *Manduca sexta* (19), *Blattella germanica* (20), and *Spodoptera litura* (21); however, two isoforms of *Ftz-f1*, namely *αFtz-f1* and *βFtz-f1*, have been detected in *Drosophila* (22) and *Leptinotarsa decemlineata* (23). *Ftz-f1* is associated with molting in *Tribolium castaneum* (24) and acts as a competence factor for 20E in the vitellogenesis of mosquitoes (18). *Ftz-f1* plays an essential role in embryogenesis, larval ecdysis, and pupation of *Drosophila melanogaster* (14, 15). In *B. germanica*, silencing of *Ftz-f1* results in molting failure and larval death (20). In vertebrates, SF1 is the key factor that regulates steroid production, and SF1 is produced by *Ftz-f1* (25). Previous studies have also shown that *Ftz-f1* regulated the expression of genes related to ecdysone biosynthesis (26). The regulation of molting-related genes may be the original function of the *Ftz-f1* protein (27, 28). In mammals, *Ftz-f1* acts as a regulator of P450 steroid hydroxylase (29). In *D. melanogaster*, the loss of *Ftz-f1* function leads to a significant decrease in the protein levels of the *disembodied* and *phantom* genes, which confirms that *Ftz-f1* has a regulatory effect on these genes (26). *Spook* and *Phantom* are the upstream gene that catalyzes the synthesis of cholesterol into 20E, and *MnFtz-f1* is the downstream gene of 20E (29). Therefore, *MnFtz-f1*, *Spook* and *Phantom* may have a synergistic effect between exercising the molting function.

Follicle maturation and ovulation are essential for successful reproduction in females. Studies have shown that *Ftz-f1* regulates the occurrence of follicles through molting signals (30). In *Drosophila*, the disruption of *Ftz-f1* expression leads to the failure of follicle cells to mature normally, eventually resulting in ovulation failure (31). Similarly, the knockdown of the *Ftz-f1* gene severely hindered yolk formation and oogenesis in *T. castaneum*, and the reproductive ability of the insect was significantly inhibited (32). The *Ftz-f1* gene also plays a role in the reproduction process of worker bees, and the size of their ovaries is regulated by *Ftz-f1* (33). After the mosquitoes have a blood meal, under the effect of 20E, *Ftz-f1* acts as a competence factor for the *Vg* gene (34). As noted above, *Ftz-f1* performs basic functions in insects, but there are fewer reports of the role of *Ftz-f1* in crustaceans. Presently, it is known that *Ftz-f1* is involved in the regulation of *Vg* in *Eriocheir sinensis* (35) and *Daphnia*, and silencing of *Ftz-f1* by interference results in molting failure. Previous studies have shown that both *MnFtz-f1* and *Vg* are related to ovarian development and may have a regulatory relationship between them.

Crustaceans are very fragile due to the lack of a protective outer shell immediately after molting (36, 37). Because of a tendency to engage in combat and autophagy, crustaceans that have just molted are vulnerable to attack by their companions. In aquaculture, abnormal molting and damage to the new epidermis layer are important reasons for the high mortality of crustaceans (38). *Macrobrachium nipponense* is a decapod crustacean with an important economic value in China’s aquaculture industry (39, 40). The abnormal molting during the annual breeding period of *M. nipponense* causes a large number of deaths, which severely restricts the development of aquaculture for this crustacean (39, 40). In addition, although the relationship between gonadal development and molting is controversial in other species, the ovarian development of *M. nipponense* is closely related to molting during the breeding period (41). Molting and ovulation are very important processes for the growth and reproduction of *M. nipponense*; however, very few studies have been conducted on the molecular mechanisms underlying these processes. Therefore, it is important to study the molecular mechanisms of molting and ovulation in *M. nipponense*. Our previous studies have summarized in detail the entire process of the Halloween gene family that catalyzes the synthesis of 20E from cholesterol and showed that the *Mn-Spook* gene plays an indispensable role in the molting process of *M. nipponense* (41). To further understand the mechanism of molting and ovulation in *M. nipponense*, the present study continued to investigate the function of the nuclear receptor gene *MnFtz-f1*.

The current study identified the nuclear receptor gene *MnFtz-f1* in *M. nipponense*. The expression of *MnFtz-f1* in different tissues and developmental stages was analyzed by quantitative real-time PCR (qRT-PCR). The 20E was administered *in vivo* to detect its effect on the expression of *MnFtz-f1*. RNAi technology was used to knock-down the expression of *MnFtz-f1* to study the regulation of *MnFtz-f1* on the *Mn*-*Spook*, *Phantom*, and *Vg* genes. After silencing of *MnFtz-f1*, ISH was performed to localize *MnFtz-f1* in the experimental and control groups, and the 20E content of *M. nipponense* was detected by ELISA. Finally, the role of *MnFtz-f1* in the molting and ovulation of *M. nipponense* was studied by comparing the molting frequency and the number of ovulations completed in the experimental and control groups.

## Results

### Molecular Cloning and Structural Analysis of the *MnFtz-f1* Gene

**Figure 1:**
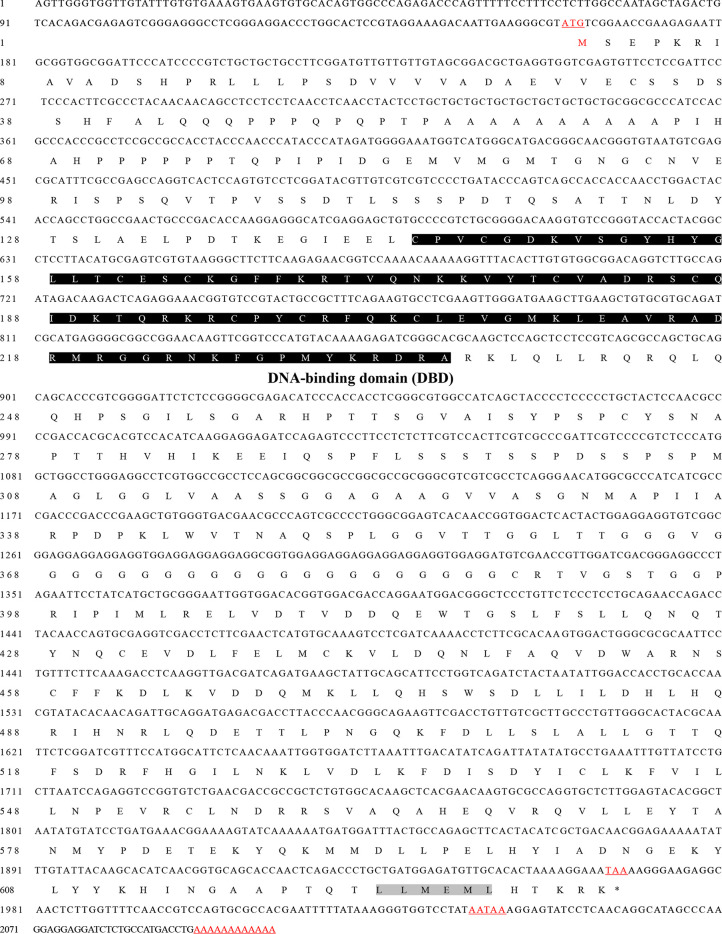
The nucleotide and amino acid sequences of the *MnFtz-f1* gene in *M. nipponense*. The numbers on the left of the sequence indicate the positions of nucleotides and amino acids. The amino acids are presented as one-letter symbols and shown below their codons in each line. The starting codon (ATG) is underlined; the termination codon (TAA) is indicated by an asterisk (^*^); and the putative polyadenylation signal (AATAAA) is underlined. The DBD domain is marked with shadow.

The full-length *MnFtz-f1* cDNA sequence was 2,198 base pairs (bp); the 5ʹ and 3ʹ noncoding regions were 160 bp and 139 bp, respectively; and the open reading frame was 1,899 bp and encoded 632 amino acids (GenBank accession number: OK217288). The *MnFtz-f1* cDNA included a polyadenylation signal (AATAAA) and a poly(A) tail in the 3ʹ-untranslated region (UTR), which indicated the integrity of the *MnFtz-f1* gene sequence (**Figure 1**).

**Figure 2:**
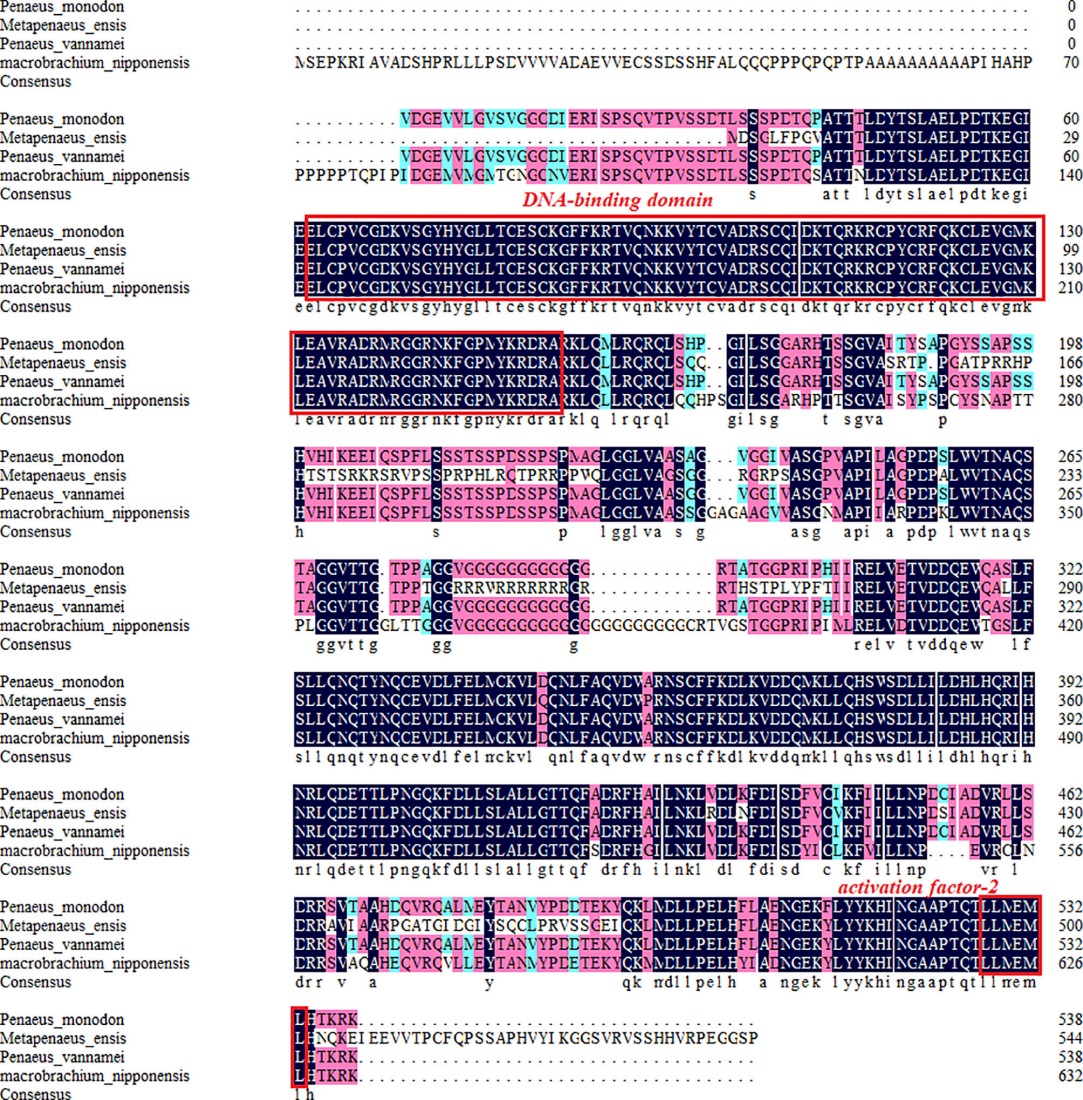
Alignment of the deduced amino acid sequence of *MnFtz-f1* with those of other species. The deduced amino acid sequence of *MnFtz-f1* in *M. nipponense* (OK217288) was compared with that of *Ftz-f1* from *P. vannamei* (QJI54417.1), *P. monodon* (XP_037803375.1), and *H. americanus* (KAG7156476.1) by the DNAMAN program.

The amino acid sequences of *MnFtz-f1* were compared with those of other crustaceans by DNAMAN 6.0. The results showed that *MnFtz-f1* had significant homology with *Ftz-f1* of other crustaceans, and both had the DNA-binding domain (DBD) and activation factor-2 (AF-2) as conserved domains. *MnFtz-f1* showed the highest amino acid identity (68.3%) with *Ftz-f1* of *Penaeus vannamei* followed by *Penaeus monodon* (68.1%) and *Homarus americanus* (50.2%) (**Figure 2**).

**Figure 3:**
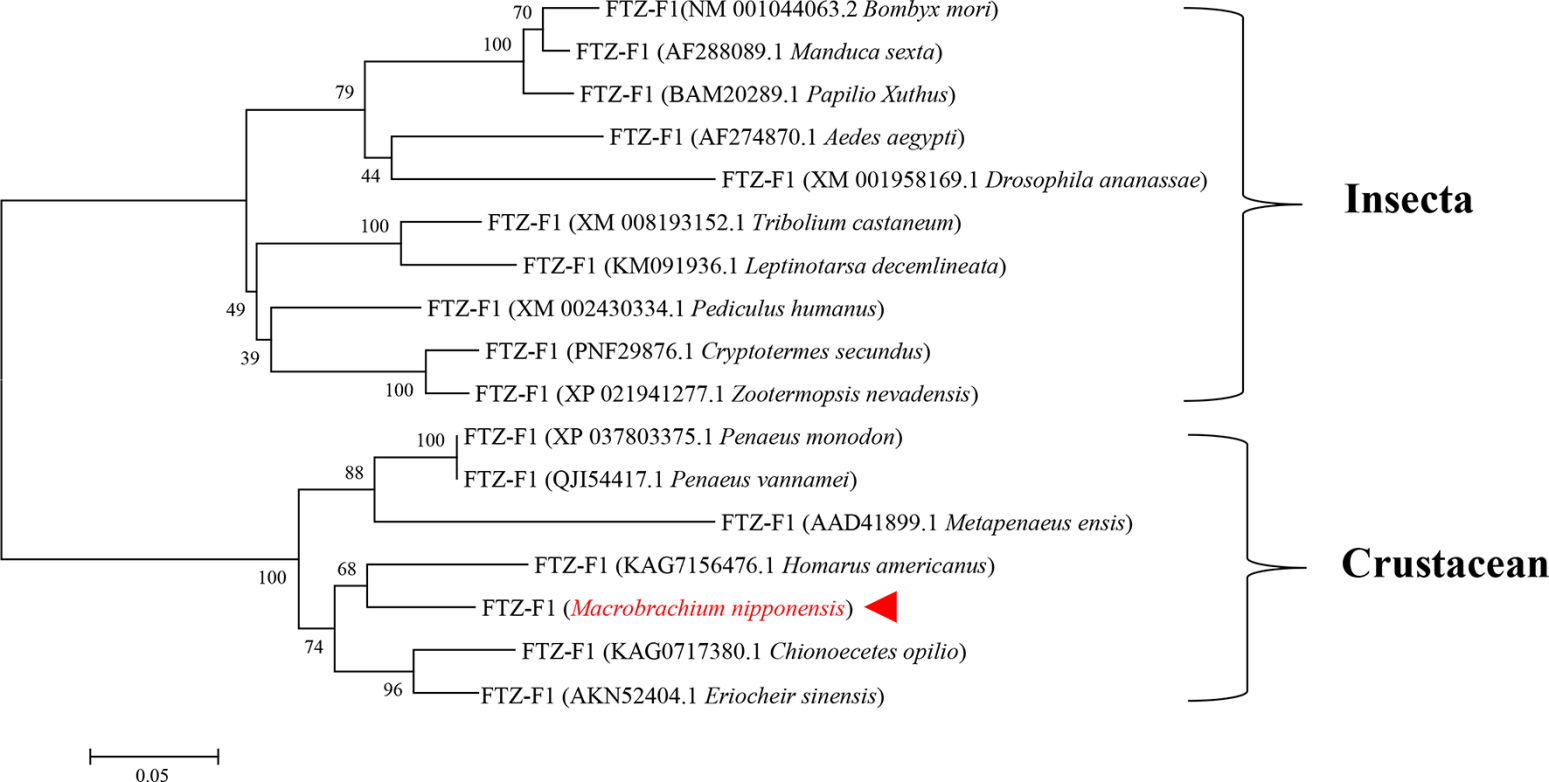
Phylogenetic tree of amino acid sequences of MnFtz-f1 from various species. GenBank accession numbers are shown in brackets. *M. nipponense* MnFtz-f1 is marked in red.

A phylogenetic tree of insects and crustaceans was constructed by MEGA 5.1 software. The results showed that the amino acid sequence of *H. americanus* clustered with the amino acid sequence of *MnFtz-f1*. The phylogenetic tree was clearly divided into two major branches, i.e., insects and crustaceans (**Figure 3**).

**Figure 4:**
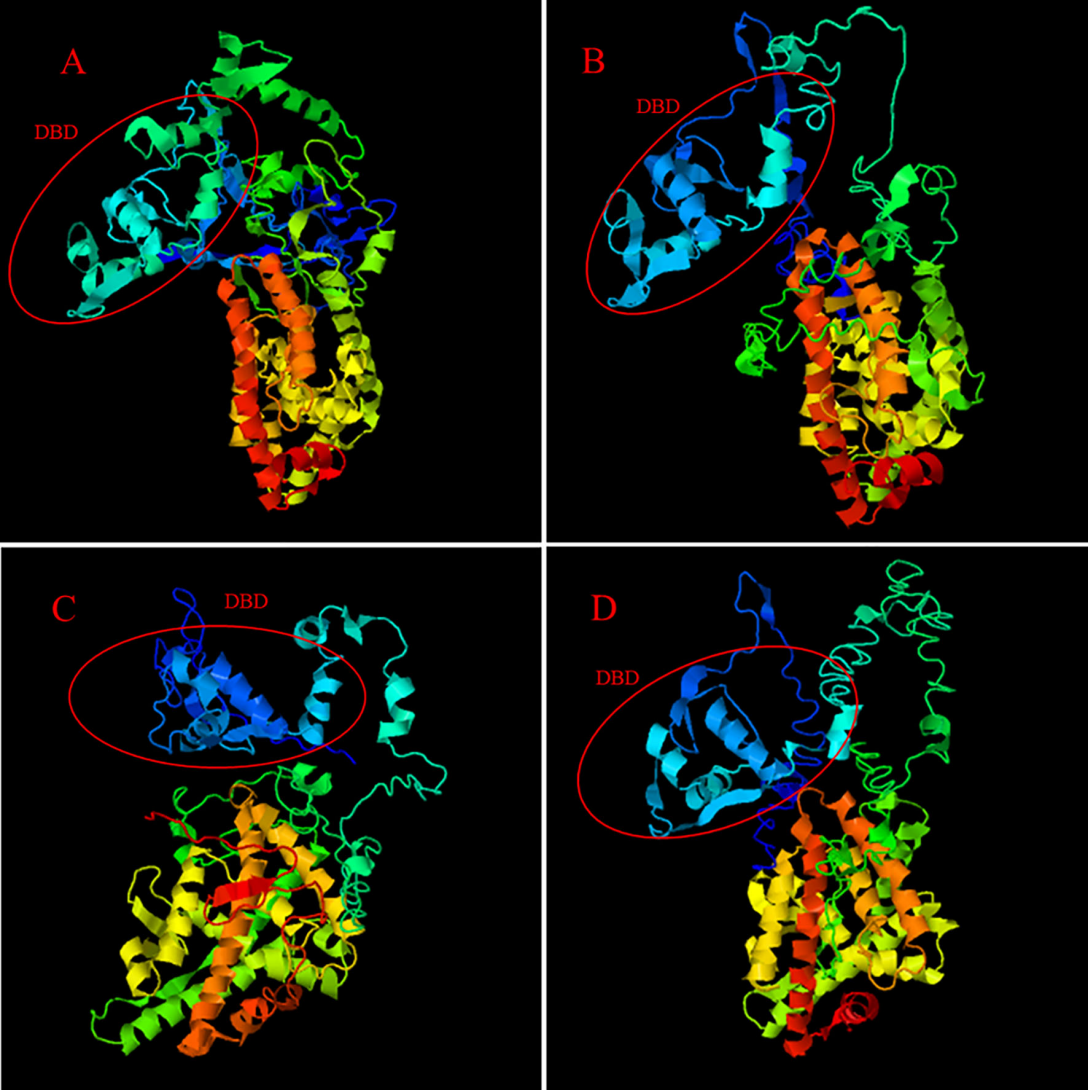
The 3D-structures of *MnFtz-f1* and *Ftz-f1* predicted by I-TASSER. **(A)** shows the 3D-structure of the *MnFtz-f1* gene of *M. nipponense*. **(B–D)** show the 3D-structures of the *Ftz-f1* gene of *P. vannamei*, *H. americanus* and *P. monodon*, respectively. The DNA-binding domain is marked with a red circle.

The iterative threading assembly refinement (I-TASSER) server (42, 43) was used to analyze and compare the Ftz-f1 amino acid sequences of *M. nipponense* and other crustaceans. The results of the three-dimensional (3D) atom model generated by I-TASSER showed that the Ftz-f1 amino acid sequences of *M. nipponense*, *P. vannamei*, and other crustaceans have the same DNA-binding domain (**Figure 4**).

### Expression of the *MnFtz-f1M* Gene in Different Tissues

**Figure 5:**
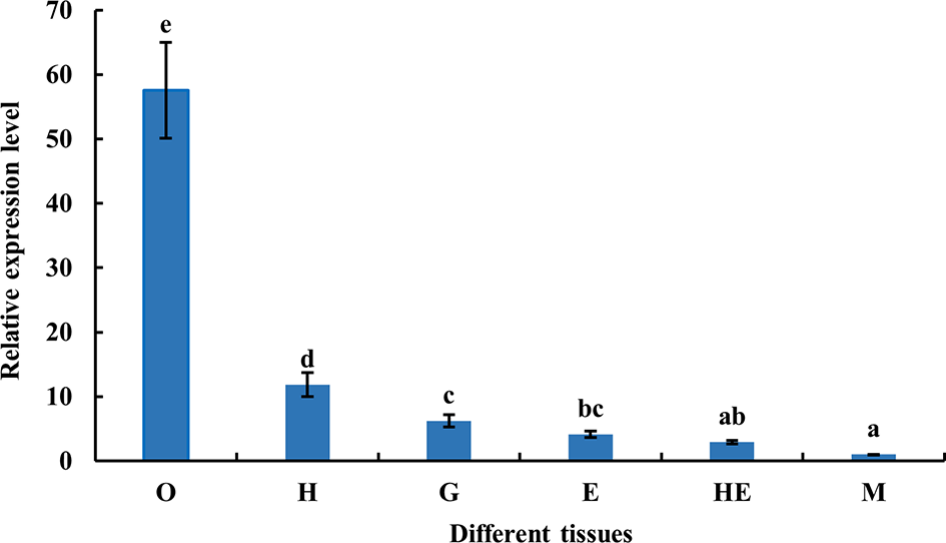
Expression of MnFtz-f1 mRNA in different tissues of *M. nipponense*. O, ovary; H, heart; G, gill; E, eyestalk; He, hepatopancreas; M, muscle. Statistical analyses were performed by one-way ANOVA. Data (mean ± SEM, n = 6) were expressed relative to the expression of the eukaryotic translation initiation factor 5A (EIF) gene. Bars with different letters indicate significant differences (P < 0.05).

The distribution of *MnFtz-f1* gene expression in different tissues (ovary, heart, gill, eyestalk, hepatopancreas, and muscle) of *M. nipponense* was determined by qPCR. As shown in **Figure 5**, the highest mRNA expression was observed in the ovary, followed by that in the heart (*P* < 0.05). The expression levels of *MnFtz-f1* in the ovary, heart and gill were 57.5-fold, 11.8-fold, and 6.2-fold higher than that in the muscle, respectively.

### Expression of the *MnFtz-f1* Gene in Different Developmental Stages of the Ovaries

**Figure 6:**
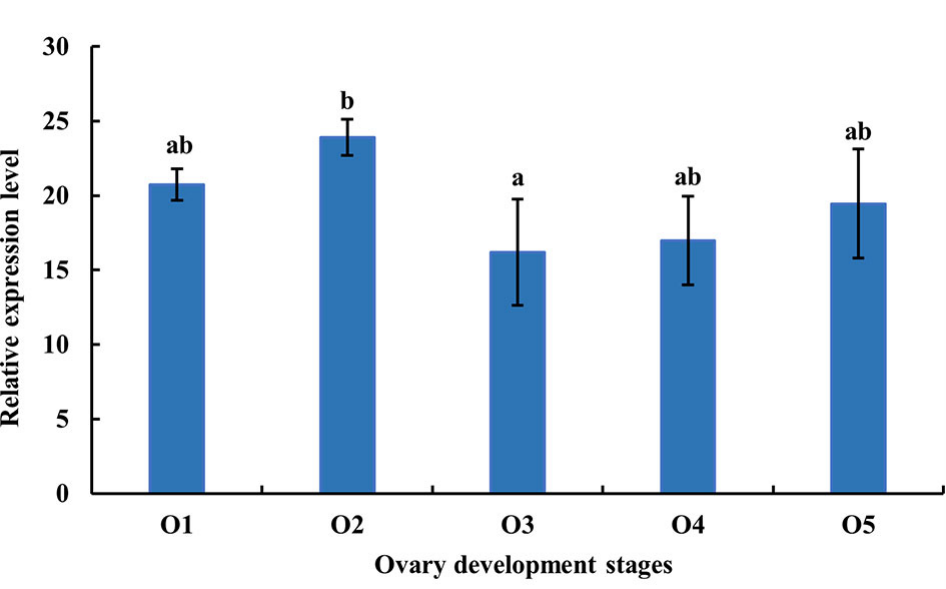
Expression of MnFtz-f1 mRNA in the developmental stages of the ovaries of *M. nipponense*. O1, undeveloped stage; O2, developing stage; O3, nearly ripe stage; O4, ripe stage; O5, spent stage. Statistical analyses were performed by one-way ANOVA. Data are expressed as mean ± SEM (n = 6). Bars with different letters indicate significant differences (P < 0.05).

As shown in **Figure 6**, the expression level of *MnFtz-f1* mRNA was the highest in the O2 stage and the lowest in the O3 stage (*P* < 0.05). There were no significant differences in the expression level of *MnFtz-f1* mRNA between the other stages of ovarian development (*P* > 0.05).

### Expression of the *MnFtz-f1* Gene in Different Developmental Stages of Embryos and Individuals

**Figure 7:**
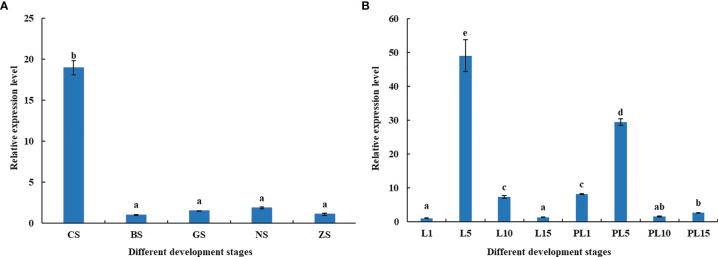
Expression of the MnFtz-f1 Gene in Different Developmental Stages of Embryos **(A)** and Individuals **(B)**. CS, cleavage stage; BS, blastula stage; GS, gastrula stage; NS, nauplius stage; ZS, zoea stage; L1, the first day after hatching; PL1, the first day after larvae, and so on. Statistical analyses were performed by one-way ANOVA. Data are expressed as mean ± SEM (n = 6). Bars with different letters indicate significant differences (*P* < 0.05).

The distribution of *MnFtz-f1* gene expression in different developmental stages was investigated by qPCR (**Figure 7**). The *MnFtz-f1* mRNA level was the highest in CS (*P* < 0.05), but no significant differences were observed between other embryonic developmental stages (BS, GS, NS, and ZS) (P > 0.05). The *MnFtz-f1* mRNA level was reached the highest on the 5th day after hatching (L5), followed by that on the 5th day after larvae (PL5) and showed significant differences with those of other developmental stages (*P* < 0.05).

### Effect of 20E on the Expression of *MnFtz-f1*

**Figure 8:**
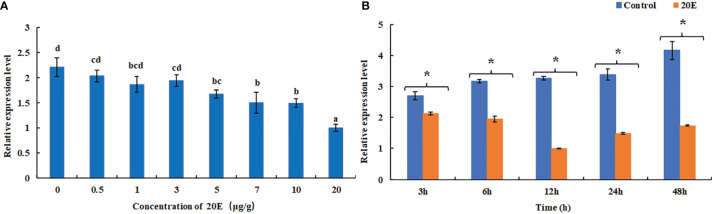
Expression of *MnFtz-f1* mRNA under the influence of different concentrations of 20E **(A)**. Effects of the same concentration of 20E (5 μg/g) on MnFTZ-F1 expression at different time points **(B)**. Statistical analyses were performed by one-way ANOVA and Student’s *t*-test. Data are expressed as mean ± SEM (n = 6). Bars with different letters and (^*^) indicate significant differences (*P* < 0.05).

The expression level of *MnFtz-f1* in the ovary under different concentrations of 20E was detected by qPCR (**Figure 8**). Compared to the control group, a low concentration of 20E (≤3 μg/g) had no significant effect on the expression of *MnFtz-f1* (*P* > 0.05). When the concentration of 20E was ≥5 μg/g, the expression of *MnFtz-f1* decreased significantly (*P* < 0.05). The expression of *MnFtz-f1* was significantly inhibited under the action of a high concentration of 20E (20 μg/g) (*P* < 0.05). The expression level of *MnFtz-f1* at different time points was detected at the same 20E concentration of 5 μg/g. The results showed that compared to the control group, the expression level of *MnFtz-f1* was significantly decreased after 20E administration (*P* < 0.05). *MnFtz-f1* expression decreased to the lowest level at 12 h and then increased gradually.

### Effect of *MnFtz-f1* Gene Knockdown on the Expression of *MnFtz-f1*, *Vg*, *Mn-Spook*, and *Phantom* in the Ovary

**Figure 9:**
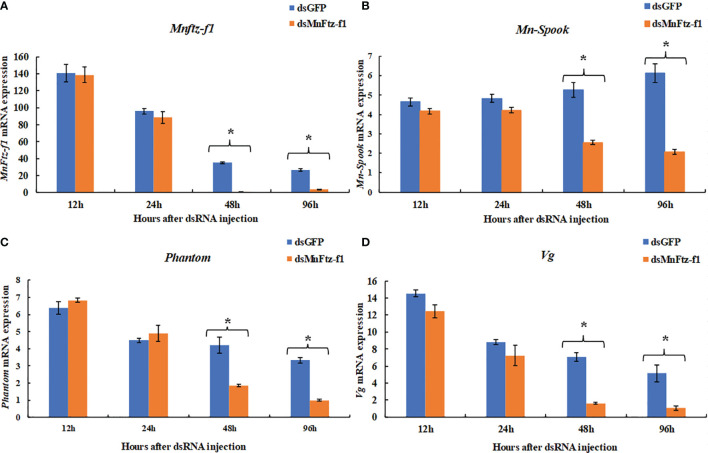
The expression levels of *Mnftz-f1*, *Mn-Spook*, *Phantom* and *Vg* after RNAi of *Mnftz-f1*. **(A)**, *MnFtz-f1*; **(B)**, *Mn-Spook*; **(C)**, *Phantom*; **(D)**, *Vg*. Data are expressed as mean ± SEM, and the differences were considered to be significant at *P* < 0.05 (^*^) by Student’s *t*-test (n = 6).

The function of *MnFtz-f1* in *M. nipponense* and its regulatory relationship with other genes were studied by the RNAi method (**Figure 9**). Compared to the control group, the expression level of *MnFtz-f1* did not decrease significantly within 24 h after ds*MnFtz-f1* RNA administration (*P* > 0.05). The expression level of *MnFtz-f1* at 48 and 96 h after the administration was significantly decreased by 97.12% and 86.09%, respectively, as compared to that of the control group (*P* < 0.05). After silencing of *MnFtz-f1*, the expression levels of *Mn-Spook*, *Phantom*, and *Vg* decreased significantly at 48 and 96 h after the administration, and the levels decreased by 51.42% and 66.06%, 56.16% and 69.82%, and 77.14% and 79.50%, respectively (*P* < 0.05).

### Effect of RNAi on the 20E Content of *M. nipponense*

**Figure 10:**
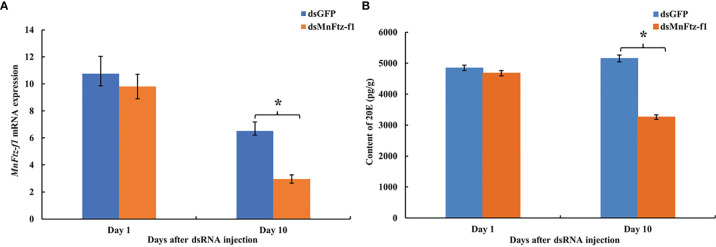
The expression level of *Mnftz-f1* **(A)** and the content of 20E **(B)** in *M. nipponense* after RNAi of *Mnftz-f1*. Data are expressed as mean ± SEM, and the differences were considered to be significant at *P* < 0.05 (^*^) by Student’s *t*-test (n = 6).

The expression level of *MnFtz-f1* on days 10 after the administration was significantly decreased by 54.70%, as compared to that of the control group (*P* < 0.05) (**Figure 10A**). The content of 20E in the ovaries of *M. nipponense* was measured by ELISA after the knockdown of *Mnftz-f1* (**Figure 10B**). Compared to the control group (dsGFP administration), the 20E content did not decrease significantly on the first day after the administration of *dsMnFtz-f1* RNA (*P* > 0.05). On the 10th day after RNAi, the content of 20E in the experimental group was significantly reduced and was 30.25% lower than that in the control group (*P* < 0.05).

### Localization of the *MnFtz-f1* Gene in the Ovaries

**Figure 11:**
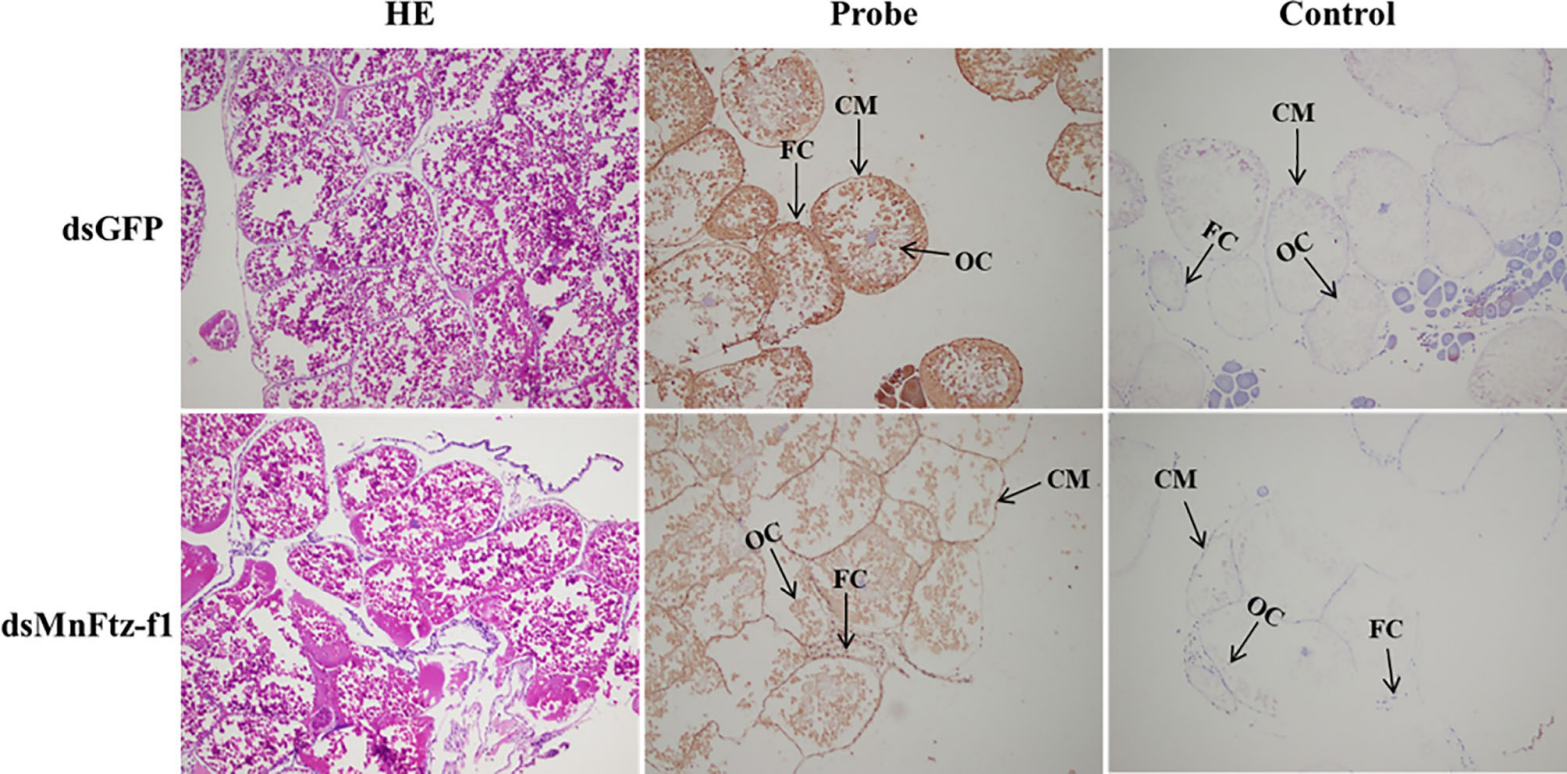
Histological sections of ovarian tissues of the experimental and control groups after RNAi. GFP was used as a control. OC, oocyte; CM, cytoplasmic membrane; FC, follicle cell; scale bar, 20 μm.

After the knockdown of the *MnFtz-f1* gene, ISH was used to label the *MnFtz-f1* mRNA in the experimental and control groups (**Figure 11**). *MnFtz-f1* signals were detected in the cytoplasmic membrane and follicular cells. Compared to the control group, the *MnFtz-f1* signals of the experimental group were weaker, and no signal was detected in the negative control.

### Effect of *MnFtz-f1* Knockdown on the Molting Frequency and Ovulation of *M. nipponense*

**Figure 12:**
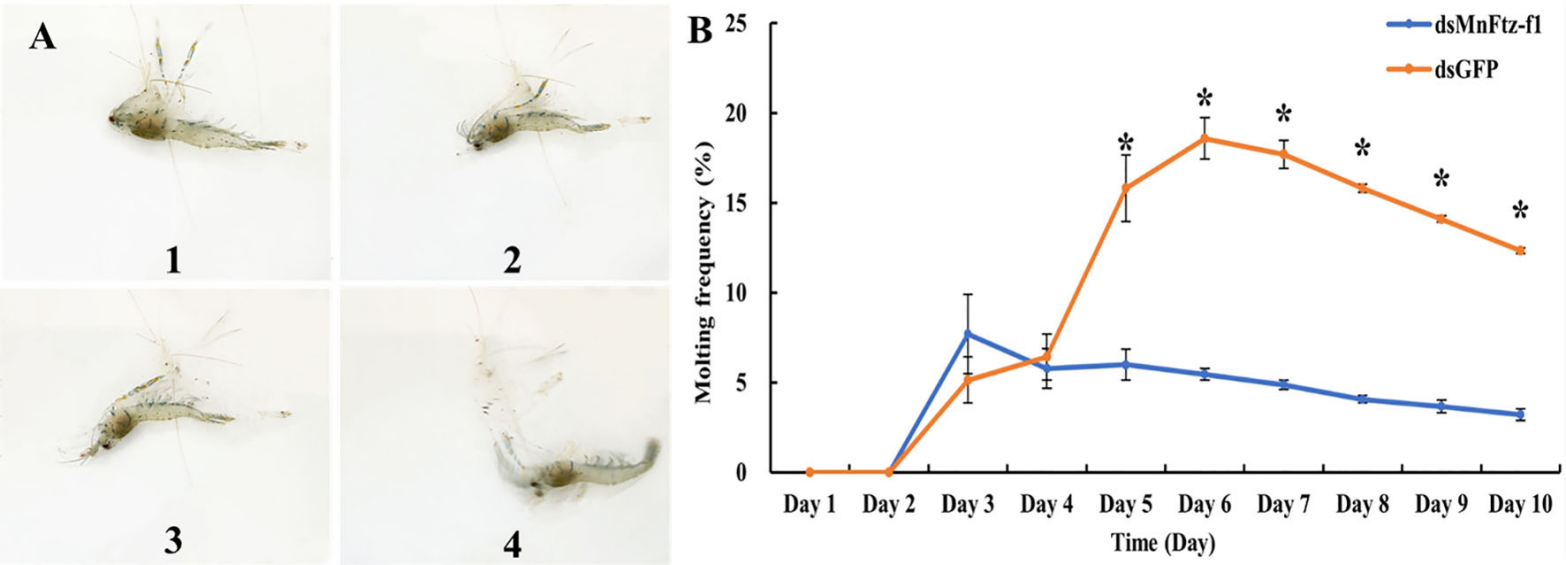
Molting frequency of *M. nipponense* in the experimental and control groups after RNAi **(B)**. The molting order of prawn was 1- 4 **(A)**. GFP was used as a control. Data are expressed as mean ± SEM, and the differences were considered to be significant at *P* < 0.05 (^*^) by Student’s *t*-test.

**Figure 12A** shows the molting process of *M. nipponense*. After *MnFtz-f1* knockdown, the molting frequency of *M. nipponense* was estimated (**Figure 12B**). The number of molting times was recorded by counting the procuticle of *M. nipponense*. *M. nipponense* began molting on the 3rd day. No significant differences were observed between the experimental and control groups on the 3rd and 4th days (*P* > 0.05). Starting from the 5th day, the molting frequency of the experimental group was significantly lower than that of the control group (*P* < 0.05).

**Figure 13:**
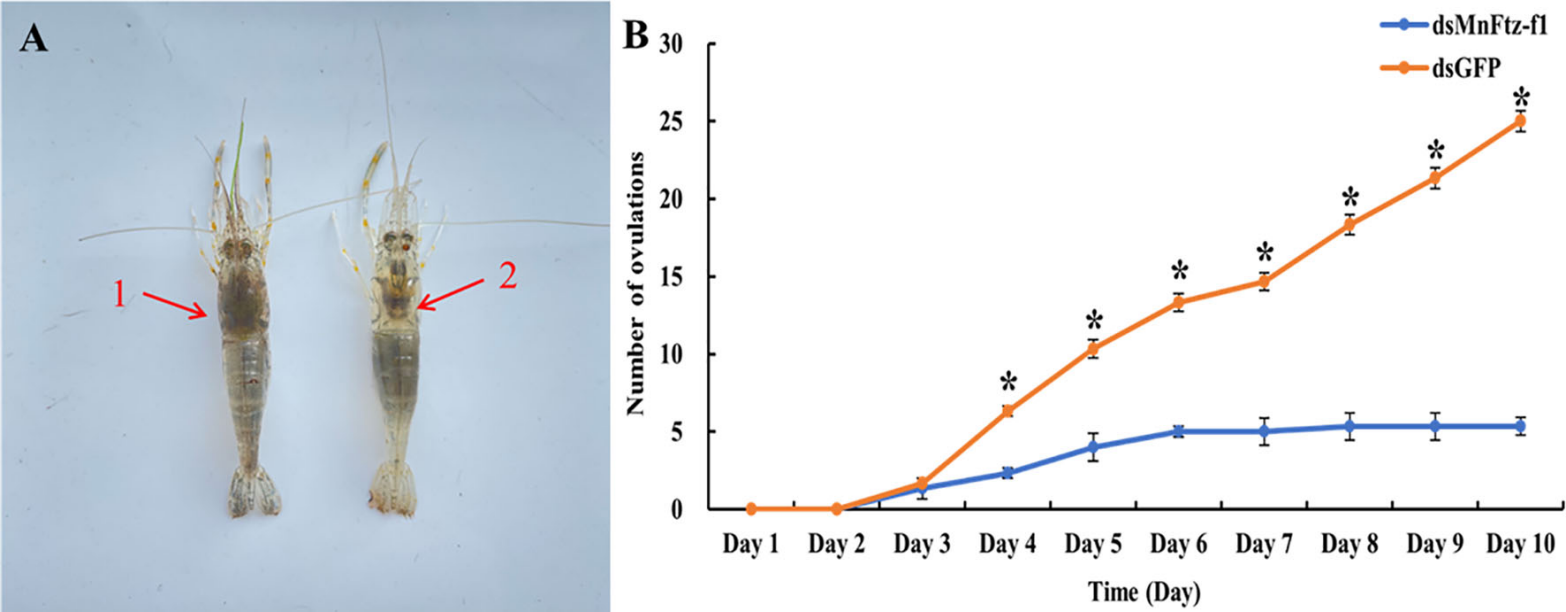
The number of ovulations of *M. nipponense* in the experimental and control groups after RNAi **(B)**. GFP was used as a control. 1, non-ovulation, 2, ovulation **(A)**. Data are expressed as mean ± SEM, and the differences were considered to be significant at *P* < 0.05 (^*^) by Student’s *t*-test.

**Figure 13A** shows the comparison of ovulation and non-ovulation of *M. nipponense*. After RNAi, we counted the number of *M. nipponense* individuals that completed ovulation in the experimental and control groups (**Figure 13B**). *M. nipponense* started ovulation on the 3rd day after interference. On the 3rd day, no significant difference in ovulation was observed between the experimental group and the control group (*P* > 0.05). From the 4th day onwards, the ovulation frequency of the experimental group was significantly lower than that of the control group (*P* < 0.05).

## Discussion

Nuclear receptor transcription factors are one of the most abundant transcription factors in metazoans, and they are involved in various developmental and physiological processes such as sex differentiation, ovarian and embryo development, and molting (44, 45). *Ftz-f1* is one of the classical nuclear receptors (46). In the present study, we focused on the orphan receptor *Ftz-f1* and successfully cloned the full-length *MnFtz-f1* cDNA from *M. nipponense* (**Figure 1**). Multiple sequence alignments indicate that *MnFtz-f1* has a nuclear receptor gene public DNA-binding domain (DBD) (10) (**Figure 2**). DBD has two Cys2-Cys2 zinc coordination modules, and subtle structural changes in DBD significantly affect transcriptional regulation (47). *MnFtz-f1* is highly conserved, especially the DBD domain. The DBD domains of *M. nipponense* are identical to those of *P. vannamei*, *H. americanus* and *P. monodon* (**Figure 2**). Phylogenetic analysis showed that crustaceans and insects were clearly delimited and clustered together (**Figure 3**), indicating that *Ftz-f1* was differentiated in crustaceans and insects and was more conserved in the same class.

In the current study, *MnFtz-f1* was found to be expressed in different tissues of *M. nipponense*, among which the expression was highest in the ovary (**Figure 5**). Similar to previous results, *Ftz-f1* has been shown to be involved in various developmental processes and is expressed in many different tissues (48). *Ftz-f1* is essential for ovarian development in *Drosophila* (49) and is also essential for oogenesis in *A. aegypti* and *T. castaneum* (18, 32). The expression of *MnFtz-f1* was highest in the ovary of *M. nipponense*, which was consistent with the finding that *Ftz-f1* plays an important role in the reproductive process (50, 51). *MnFtz-f1* expression in the different developmental stages of *M. nipponense* ovary did not show alterations with the development of the ovary; however, the expression level was the lowest in the O3 stage, and this level was significantly lower than that in the O2 stage (**Figure 6**). *MnFtz-f1* expression in the O3 stage may be inhibited by 20E, which has been shown to significantly inhibit the expression of *Ftz-f1* (16). When the concentration of 20E drops to a low level, the expression of *Ftz-f1* initially inhibited by 20E begins to increase (48, 52–55). The embryonic stage is a special life stage with no food intake and no activity. Therefore, genes that are highly expressed at this stage are directly involved in embryonic development or in preparing for future physiological stages (56). The expression of *MnFtz-f1* in the CS of *M. nipponense* was significantly higher than that in the other developmental stages (**Figure 7**); this showed that *MnFtz-f1* might play an important role in the process of oocyte mitosis. A recent study in *Drosophila* revealed that *Drosophila* oocytes could not undergo normal mitosis in the absence of *Ftz-f1*, suggesting that *Ftz-f1* was essential for oocyte division (57). In *Drosophila*, *Ftz-f1* is divided into two subtypes: *αFtz-f1* and *βFtz-f1*. The *αFtz-f1* is mainly expressed in the early stage of embryogenesis, while *βFtz-f1* is expressed in the late embryonic stage and pupal stage (58). In the current study, *MnFtz-f1* was highly expressed in the early stage of major embryogenesis (CS), on the 5th day after hatching, and on the 5th day after larvae (**Figure 7**). *MnFtz-f1* may have a similar function of *αFtz-f1* and *βFtz-f1* in the embryonic and hatching stages. *Ftz-f1* is one of the 20E responsive genes, and the decrease in 20E level induces an increase in *βFtz-f1* expression level (15, 17, 59). Consistent with previous research, *in vivo* administration of 20E significantly inhibited the expression level of *MnFtz-f1* (**Figure 8**).

RNAi causes post-transcriptional gene silencing through double-stranded RNA (dsRNA) (60). In *M. nipponense*, RNAi has been widely used in gene function analysis (41, 61, 62). In the current study, the expression of *MnFtz-f1* in *M. nipponense* ovaries was significantly reduced by the *in vivo* administration of dsRNA. To further study the mutual relationship of regulation between the genes, the expression levels of *Mn-Spook*, *Phantom*, and *Vg* were determined after *MnFtz-f1* knockdown. *Spook* and *Phantom* are important members of the Halloween gene family and regulate molting by catalyzing the conversion of cholesterol to 20E (3). *Mn-Spook* plays a pivotal role in the molting of *M. nipponense* by participating in 20E production (41). In *Schistocerca gregaria*, silencing of *Spook* reduces ecdysteroid titer and leads to delayed nymphal development and failure to molt. *Phantom* is the enzyme required by the prothoracic glands of *Bombyx* and *Drosophila* to synthesize ecdysteroid (63). In crustaceans, *Vg* provides energy for ovarian development, and the maturation of ovaries depends on the synthesis and accumulation of *Vg* (64, 65). In general, *Mn-Spook*, *Phantom*, and *Vg* are closely related to the molting or ovarian development of crustaceans. Studying the regulatory relationship between *MnFtz-f1* and these genes in *M. nipponense* is more conducive to our understanding of the molting and ovarian development processes of *M. nipponense* at the molecular level. In the current study, the expression levels of the *Mn-Spook*, *Phantom*, and *Vg* genes were also significantly reduced after silencing of *MnFtz-f1* (**Figure 9**). Previous studies have shown that *Ftz-f1* could regulate the expression of the Halloween genes and affect the ecdysone titer (26, 66). In the *Drosophila* ring gland, *Ftz-f1* mutation caused a significant decrease in the expression level of *Phantom*, indicating that *Ftz-f1* regulated the expression of *Phantom* (26). In *T. castaneum*, silencing the expression of *Ftz-f1* results in a complete decrease in the expression of the *Vg* gene (32). *Ftz-f1* plays a key role in the regulation of *Vg* in *A. aegypti* (30). In *Apis mellifera*, RNAi experiments showed that *Ftz-f1* regulates the expression of *Vg* (51). In summary, our research confirmed that *MnFtz-f1* regulated the expression of *Mn-Spook*, *Phantom*, and *Vg*. RNAi of *MnFtz-f1* significantly reduced the content of 20E in *M. nipponense* (**Figure 10**). Similar to our results, *Ftz-f1* plays a role in regulating ecdysone titer during the development of *D. melanogaster* (26, 67). Our results strongly confirmed that high concentrations of 20E inhibited the expression of *MnFtz-f1*, but knockdown *MnFtz-f1* inhibited the expression of the *Mn-spook* and *Phantom* genes involved in the synthesis of 20E, thereby affecting the efficiency of 20E synthesis. Therefore, we speculated that *MnFtz-f1* played a role of negative feedback regulation during the synthesis of 20E. The results of ISH showed that more *MnFtz-f1* signals were detected in the oocyte plasma membrane and follicular cells, and more *MnFtz-f1* signals were detected in the control group than in the experimental group (**Figure 11**). Similarly, *Ftz-f1* was detected in the follicular cells of the ovary of *D. melanogaster* (68).

To determine whether *MnFtz-f1* played a role in the molting and ovulation of *M. nipponense*, we estimated the molting frequency and ovulation number of *M. nipponense* after *MnFtz-f1* knockdown. The results showed that the molting and ovulation of *M. nipponense* in the experimental group were significantly inhibited as compared to that in the control group (**Figures 12** and **13**). Similar studies in insects have shown that *Ftz-f1* played a role in molting and ovarian development. In *L. decemlineata*, knockdown of *Ftz-f1* causes surface defects in wings and legs and disrupts molting (23). Several studies have shown that silencing of *Ftz-f1* could lead to failure of larvae to undergo pupation and molting (20, 24, 48, 69). Similar to our results, the role of *Ftz-f1* in ovulation was also demonstrated in *Drosophila*. In *Drosophila*, *Ftz-f1* promotes follicle maturation and ovulation. The interruption of *Ftz-f1* expression prevents follicle maturation and causes ovulation failure (31). In *B. germanica*, *Ftz-f1* knockdown leads to severe obstruction of ovulation (50), while *Drosophila* requires *Ftz-f1* to promote ovulation in the final stage. Other studies have also shown that *Ftz-f1* is essential for the oogenesis of *A. aegypti* (18) and *T. castaneum* (32).

In conclusion, we identified the nuclear receptor gene *MnFtz-f1* in *M. nipponense*. The expression, distribution, and function of the *MnFtz-f1* gene in *M. nipponense* were systematically analyzed by qRT-PCR, RNAi, ISH, ELISA, and other techniques. The results of the present study strongly confirmed that *MnFtz-f1* played a pivotal role in the molting and ovulation processes of *M. nipponense*. This study enriched the molecular mechanisms of molting and ovulation during the reproduction period of *M. nipponense* and provided new insights for studying the relationship between molting and ovarian development in crustaceans.

## Materials and Methods

### Ethics Statement

All experimental animals (*M. nipponense*) in this study were handled according to the guidelines of the Institutional Animal Care and Use Ethics Committee of the Freshwater Fisheries Research Center, Chinese Academy of Fishery Sciences (Wuxi, China).

### Animals

Healthy adult female prawns (2.19 ± 0.66 g) were obtained from the Freshwater Fisheries Research Center, Chinese Academy of Fishery Sciences (120^◦^13′44′′E, 31^◦^28′22′′N). The prawns were cultured in circulating water (26°C ± 1°C), and snails were fed twice a day. The experiment was conducted after 1 week of acclimatization.

### RNA Isolation and cDNA Synthesis From Tissue

According to the manufacturer’s protocols, the RNAiso Plus kit (TaKaRa, Japan) was used to extract total RNA from the whole tissues of prawns (n=6). The quality of RNA was determined by 1.2% agarose gel. NanoDrop ND2000 (NanoDrop Technologies, Wilmington, DE, USA) was used to determine the concentration and purity of RNA, and the ratio of A260/A280 was estimated to determine the integrity of RNA. DNase I (Sangon, Shanghai, China) was used to process RNA samples to eliminate possible DNA contamination. The first-strand cDNA was synthesized using the reverse transcriptase M-MLV kit (TaKaRa). The synthesized cDNA was stored at -80°C for further experiments.

### Cloning and Bioinformatics Analysis of *MnFtz-f1*

The cDNA fragment of the target gene *MnFtz-f1* was obtained from the *M. nipponense* transcriptome cDNA library (ID: PRJNA533885) in our laboratory. The 3′-full RACE Core Set Ver. 2.0 kit and the 5′-full RACE kit (TaKaRa) were used to clone 3′-cDNA and 5′-cDNA according to the manufacturer’s protocols, respectively. Based on the known cDNA fragments, specific primers for *MnFtz-f1* were designed for full-length cloning of the *MnFtz-f1* cDNA. An automated DNA sequencer (ABI Biosystems, USA) was used to verify the nucleotide sequence of the cloned cDNA. All primers were synthesized by Shanghai Sangon Biotech Company (Shanghai, China) (**Table 1**). DNAMAN 6.0 was used to assemble the full length of the *MnFtz-f1* cDNA. The *MnFtz-f1* gene sequence was analyzed using GenBank BLASTX and BLASTN programs (http://www.ncbi.nlm.nih.gov/BLAST/). The online program ORF Finder (http://www.ncbi.nlm.nih.gov/gorf/gorf.html) was used to analyze the open reading frame of the *MnFtz-f1* gene. Phylogenetic trees based on the amino acid sequences were generated by the neighbor joining method with Molecular Evolutionary Genetics Analysis (MEGA5.0) software, and the bootstrapping replications were 1,000 (70, 71). Multiple sequence alignment of *MnFtz-f1* amino acids was performed using DNAMAN 6.0 software. The spatial structure was predicted by I-TASSER (https://zhanglab.ccmb.med.umich.edu/I-TASSER/). The amino acid sequences of other arthropods investigated in this study were downloaded from the GenBank database (http://www.ncbi.nlm.nih.gov/).

**Table 1:** Primers used in this study.

Primer Name	Sequence(5′-3′)	Usage
5′-RACE outer	GAGACGACCTTACCCAACGG	For 5′-RACE
5′-RACE inner	CTTGTTCGTGAGCTTGTGCC	For 5′-RACE
3′-RACE outer	CTCCGATTCCTCCCACTTCG	For 3′-RACE
3′-RACE inner	ACGACGACAACGTATCCGAG	For 3′-RACE
*MnFtz-f1-F*	CCTACAACCAGTGCGAGGTC	For 3′-RACE
*MnFtz-f1-R*	TCCGAGAATTGCGTAGTGCC	For 3′-RACE
*MnFtz-f1-*qF	GCAAAGTCCTCGATCAAAACCTC	Primer for *MnFtz-f1* expression
*MnFtz-f1-*qR	GAAACGATCCGAGAATTGCGTAG	Primer for *MnFtz-f1* expression
*Mn-Spook*-qF	CCTATGCGACTACTCTGAACTCC	Primer for *Mn-Spook* expression
*Mn-Spook*-qR	TCTGGAAGGTCTTGTTGTCGTAG	Primer for *Mn-Spook* expression
*Mn-Vg*-qF	GAAGTTAGCGGAGATCTGAGGT	Primer for *Mn-Vg* expression
*Mn-Vg*-qR	CCTCGTTGACCAATCTTGAGAG	Primer for *Mn-Vg* expression
*Mn-Phantom*-qF	ATACGGTCTGATATGCTCCGATG	Primer for *Mn- Phantom* expression
*Mn-Phantom*-qR	GGGTATTTCCTCCCGAAGATGAG	Primer for *Mn- Phantom* expression
EIF-F	TATGCACTTCCTCATGCCATC	Primer for EIF expression
EIF-R	AGGAGGCGGCAGTGGTCAT	Primer for EIF expression
*MnFtz-f1* Probe	ACACTGGAGTGACCTGGCTCGGCGAAATGC	Probe for *MnFtz-f1* ISH analysis
*MnFtz-f1* control	GCATTTCGCCGAGCCAGGTCACTCCAGTGT	Probe for *MnFtz-f1* ISH analysis
GFP -iF	TAATACGACTCACTATAGGGACGAAGACCTTGCTTCTGAAG	For GFP dsRNA
GFP -iR	TAATACGACTCACTATAGGGAAAGGGCAGATTGTGTGGAC	For GFP dsRNA
*MnFtz-f1-*iF	TAATACGACTCACTATAGGGGCTCGATCAAAACCTCTTCGC	For *MnFtz-f1* dsRNA
*MnFtz-f1*-iR	TAATACGACTCACTATAGGGGACATCTCCATCAGCAGGGTC	For *MnFtz-f1* dsRNA

### The qRT-PCR Analysis

The Bio-Rad iCycler iQ5 Real-Time PCR System (Bio-Rad, Carlsbad, CA, USA) was used to perform the SYBR Green qRT-PCR assay. The reaction system and procedures of qRT-PCR were consistent with our previous study (41). *MnEIF* was used as the internal control gene (72). All primers used for qRT-PCR are listed in **Table 1**. The expression level of all genes in this experiment was calculated by the 2^-ΔΔCt^ method (73). The ovarian development cycle was classified into different stages according to previous studies (74) as follows: O1 (undeveloped stage, transparent), O2 (developing stage, yellow), O3 (nearly ripe stage, light green), O4 (ripe stage, dark green), and O5 (spent stage, gray). All experiments were performed in triplicate for each group, with at least five samples in each group.

### ISH

The localization of *MnFtz-f1* mRNA was determined by ISH, and the detailed steps are described in Li et al. (75). According to the *MnFtz-f1* cDNA sequence, the probe was designed with Primer5 software (http://www.premierbiosoft.com/primerdesign/). ISH experiments were performed in triplicate for each tissue, and the results were evaluated under a light microscope.

### Effect of 20E on *MnFtz-f1*

On the basis of previous reports (76–78), 20E (Sigma-Aldrich, USA) with different concentration gradients (0.5, 1, 3, 5, 7, 10, and 20 µg/g) was administered through injection into prawns, and tissues were collected after 3 h to detect the expression level of *MnFtz-f1*. The same volume of ethanol was administered to the control group (0 µg/g). A fixed concentration based on the results of the 20E concentration experiment was selected and administered into *M. nipponense* to test its effect on the expression of *MnFtz-f1* at different time points (3, 6, 12, 24, and 48 h). Six prawn tissues were collected in each group in triplicate. The collected tissues were rapidly frozen in liquid nitrogen and stored in a refrigerator at -80°C until mRNA extraction.

### RNA Interfering

*MnFtz-f1* primers and the Green Fluorescent Protein (GFP) gene were designed for RNAi using Snap Dragon tools (https://www.flyrnai.org/cgi-bin/RNAi_find_primers.pl). GFP was used as a control. The dsRNA was synthesized by the AidTMT7 High Yield Transcription Kit (Fermentas Inc., Waltham, MA, USA) according to the manufacturer’s instructions. The integrity and purity of dsRNA were detected by 1.2% agarose gel electrophoresis. A total of 300 healthy female prawns (2.19 ± 0.66 g) were randomly divided into the experimental group and the control group in triplicate (n=50). According to the previous 20E injection concentration, the experimental group was administered with *MnFtz-f1* dsRNA, and the control group was administered with GFP (79) (4 µg/g of body weight). To prolong the interference efficiency of RNAi, dsRNA was administered every 5 days. Six prawns were randomly collected from each group at 12, 24, 48, and 96 h after injection, rapidly frozen with liquid nitrogen, and stored in a refrigerator at -80°C until mRNA extraction (n = 6). By silencing the *MnFtz-f1* gene, we calculated the molting frequency (MF) and ovulation of *M. nipponense*. In addition, 180 prawns (O4) were divided into the experimental and control groups in triplicate to observe the number of molting and ovulation (n = 30). MF = (Nm/Ns)/D, where Nm is total molting times; Ns is the number of prawns in aquarium; and D is experimental days (80).

### ELISA

After silencing the *MnFtz-f1* gene, the ovaries of the experimental and control groups were collected on the 1st and 10th day to detect the content of 20E. As reported earlier (41), the Shrimp EH ELISA Kit (Lot number: E20210925-98502B; Meibo, Shanghai, China) was used to detect the content of 20E in the ovaries.

### Statistical Analysis

All quantitative data conformed to homogeneity of variance and normal distribution and are expressed as mean ± standard error of the mean (SEM). Statistical analyses were performed using SPSS 20.0 software (IBM, New York, NY, USA). One-way ANOVA was used to analyze the differences in tissue distribution and different developmental stages. A two-sided *t*-test was used to compare the expression levels in the RNAi analysis. *P* < 0.05 was considered to be statistically significant.

## Data Availability Statement

The original contributions presented in the study are included in the article/supplementary material. Further inquiries can be directed to the corresponding authors.

## Ethics Statement

The animal study was reviewed and approved by Institutional Animal Care and Use Ethics Committee of the Freshwater Fisheries Research Center, Chinese Academy of Fishery Sciences (Wuxi, China).

## Author Contributions

HQ and HF: designed the study. HY: carried out the experiments and wrote the original draft. WZ and YF: provided technical assistance. HY and SZ: participated in methodology and data curation. YG, SJ, and YX: compiled resources. YW: performed software analysis. All authors contributed to the article and approved the submitted version.

## Funding

This study was supported by grants from the National Key R&D Program of China (2018YFD0901303); Central Public-interest Scientific Institution Basal Research Fund CAFS (2020TD36); Jiangsu Agricultural Industry Technology System; the New cultivar breeding Major Project of Jiangsu province (PZCZ201745); the China Agriculture Research System-48 (CARS-48).

## Conflict of Interest

The authors declare that the research was conducted in the absence of any commercial or financial relationships that could be construed as a potential conflict of interest.

## Publisher’s Note

All claims expressed in this article are solely those of the authors and do not necessarily represent those of their affiliated organizations, or those of the publisher, the editors and the reviewers. Any product that may be evaluated in this article, or claim that may be made by its manufacturer, is not guaranteed or endorsed by the publisher.

## Abbreviations

*M. nipponense, Macrobrachium nipponense*; *MnFtz-f1, Macrobrachium nipponense Ftz-f1*; 20E, 20-hydroxyecdysone; RNAi, RNA interference; ELISA, Enzyme Linked Immunosorbent Assay; qPCR, quantitative real-time reverse transcription PCR; ORF, open reading frame; O, ovary; H, heart; G, gill; E, eyestalk; HE, hepatopancreas; M, muscle; O1, undeveloped stage; O2, developing stage; O3, nearly-ripe stage; O4, ripe stage; O5, spent stage; CS, cleavage stage; BS, blastula stage; GS, gastrula stage; NS, nauplius stage; ZS, zoea stage; L1, the first day after hatching; PL1, the first day after larvae; OC, oocyte; CM, cytoplasmic membrane; FC, follicle cell; cDNA, complementary DNA; ISH, *in situ*, hybridization; Nm, total molting times; Ns, number of prawns in water aquarium; D, experimental days.
